# Promoter hypermethylation of *MGMT* gene may contribute to the pathogenesis of gastric cancer: A PRISMA-compliant meta-analysis

**DOI:** 10.1097/MD.0000000000006708

**Published:** 2017-04-28

**Authors:** Zongxin Zhang, Shaojun Xin, Min Gao, Yunxiang Cai

**Affiliations:** a Department of Clinical Laboratory, Huzhou Central Hospital; b Department of Clinical Laboratory, The First People's Hospital of Huzhou, Huzhou, Zhejiang, China.

**Keywords:** meta-analysis, MGMT, promoter hypermethylation, stomach neoplasms

## Abstract

**Beckground:**

The association of MGMT (O^6^-methyguanine deoxyribonucleic acid methyltransferase) promoter hypermethylation with gastric cancer (GC) risk has been studied extensively, but the results remained unclear. Here, we performed a meta-analysis to evaluate whether promoter hypermethylation of the *MGMT* gene contributed to gastric pathogenesis.

**Methods:**

Relevant studies were identified by retrieving the PubMed, Web of Science, Embase, and China National Knowledge Infrastructure (CNKI) databases. The Newcastle–Ottawa Scale was applied to assess methodological quality of the included studies. Pooled odds ratios (ORs) and 95% confidence intervals (95% CIs) were calculated to evaluate the association of MGMT promoter hypermethylation with gastric pathogenesis. Moreover, STATA 12.0 software was used to summarize the extracted data in this meta-analysis.

**Results:**

Seventeen studies, comprising 1736 cases and 1291 controls, were included in this meta-analysis. The frequency of *MGMT* promoter hypermethylation in the GC group (32.97%) was significantly higher than those in the control group (18.00%) (OR = 2.83, CI = 1.93–4.15, *P* < .05). When stratified by cancer subtype, the results indicated that the frequency of MGMT promoter hypermethylation was significantly higher in gastric adenocarcinoma than in control group (OR = 3.47, CI = 1.06–11.35, *P* < .05). In addition, *MGMT* promoter hypermethylation significantly promoted distant metastasis and lymph node (LN) metastasis of gastric tumor (for distant metastasis, OR = 4.22, CI = 2.42–7.37, *P* < .05; for LN metastasis, OR = 1.56, CI = 1.14–2.13, *P* < .05). A significant association between MGMT promoter hypermethylation and TNM-stage was also found in the present meta-analysis (OR = 2.70, CI = 1.79–4.08, *P* < .05).

**Conclusion:**

The results of this meta-analysis suggested that *MGMT* gene-promoter hypermethylation was significantly associated with an increased risk of GC, especially in Asians. Furthermore, *MGMT* gene-promoter hypermethylation might be correlated with the distant metastasis and LN metastasis of GC.

## 1 Introduction

Gastric cancer (GC) is one of the most common malignancies and remains the second most common cause of cancer-related death worldwide.^[1]^ It accounts for approximately 70% new cases that occur in developing countries.^[2]^ Although advances of early detection have led to a decline in incidence rates of GC, a lot of GC patients were still diagnosed at late stage, and patients usually died of metastasis.^[3]^ Many studies have confirmed that the development of GC was a multifactorial process that involved some environmental factors, multiple genetic, and epigenetic alterations such as *Helicobacter pylori* (*H. pylori*) infection, high salt intake, smoking, gene variation, and gene methylation.^[4,5]^ In terms of risk evaluation, *H. pylori* infection was significantly associated with the risk of GC. However, *H. pylori* infection was insufficient for predicting the risk of GC. Thus, other biomarkers were still needed to be identified to improve our understanding of gastric carcinogenesis.^[6]^ Epigenetic changes such as promoter methylation and histone acetylation played an important role in cancer development. A number of tumor-related genes were often silenced by those deregulated modifier that produced epigenetic changes.^[7]^ Most deoxyribonucleic acid (DNA) methylation events usually existed in the CpG dinucleotides and especially in gene promoters.^[8]^ DNA methylation was a major cause of gene silencing and normally appeared in X-chromosome inactivation, imprinted genes, and tumor-suppressor genes.^[9]^ For instance, mutation of *CDH1* gene was the main cause of hereditary diffuse GC, and DNA methylation often occurred to inactivate this gene.^[10]^ Therefore, DNA methylation might be considered as a good marker to assess the tumorigenesis, which was also beneficial for cancer diagnosis and treatment.

O^6^-methyguanine DNA methyltransferase (MGMT), a DNA repair protein encoded by *MGMT* gene that located at 10q26, removed cytotoxic and mutagenic adducts from the O^6^-guanine of DNA.^[11]^ Alkylation at the O^6^ position of guanine in DNA contributed a lot to the emergence of gene variation in cancers, because thymine tended to replace cytosine to pair with O^6^-methylguanine in the replication of DNA, which eventually resulted in the G > A mutation. MGMT protein could protect DNA from methylation damages, thus it had the ability of tumor inhibition. In contrast, it might produce chemoresistance to anticancer treatment, therefore leading to treatment failure.^[12]^ The loss of MGMT expression was mainly due to epigenetic event other than the deletions or rearrangements of the gene.^[13]^ The hypermethylation of the CpG islands on *MGMT* gene promoter significantly silenced the *MGMT* gene.^[14]^ In recent studies, significant associations between MGMT promoter hypermethylation and glioblastoma, breast cancer, and colorectal cancer were found. Many studies evaluating the correlation of MGMT promoter hypermethylation with GC were also performed. However, limitations of sample type, sample size, and race lowered the reliability of the results. Hence, in order to clarify the association between GC pathogenesis and MGMT promoter hypermethylation, we performed this meta-analysis.

## 2 Material and methods

### 2.1 Literature search strategy

PubMed, Web of Science, Embase, and CNKI databases were searched up to September 2016 using the search terms “Stomach Neoplasms[Mesh],” “gastric cancer,” “gastric tumor,” “gastric carcinoma,” “MGMT protein, human” [supplementary concept], “MGMT,” and “‘Methylation’[Mesh].” The references of included articles and relevant review literatures were searched and scanned to acquire additional eligible studies. In this retrospective meta-analysis, institutional review board approval was not required.

### 2.2 Inclusion criteria and exclusion criteria

Published studies included must meet the following criteria: studies that evaluated the association of MGMT promoter methylation with GC risk or clinical characteristics, studies that provided sufficient data on the promoter methylation of MGMT in control group and GC group, and all cancer patients were diagnosed according to the diagnostic criteria of GC. If studies did not meet these inclusion criteria, they were excluded.

### 2.3 Data extraction

Two investigators independently extracted the relevant data from included studies according to the inclusion criteria. Any discrepancy was discussed and settled by consulting with the team. The following information were collected in this meta-analysis: name of the first author, publication year of article, country of subjects, ethnicity of subjects, the number of controls and cases, GC type, clinical information, detection method of methylation, and sample type.

### 2.4 Methodological assessment

Two investigators independently assessed methodological quality of included articles using the Newcastle–Ottawa Scale (NOS) (http://www.ohri.ca/programs/clinical_epidemiology/oxford.asp) table. The NOS criteria included 3 parts: subjects selection, 0 to 4 scores; comparability of subjects, 0 to 2 scores; and exposure of subjects, 0 to 3 scores. The scores range from 0 to 9, and a score of ≥7 indicated a high-quality study.

### 2.5 Statistical analysis

In order to evaluate the strength of the association between *MGMT* gene-promoter hypermethylation and GC pathogenesis, the pooled odds ratios (ORs) and 95% confidence intervals (95% CIs) were calculated using STATA 12.0 software (Stata Corp LP, College Station, TX) software. The degree of heterogeneity present in different studies was performed based on Cochran *Q* statistic and *I*^2^ tests. When a *P* value of <.05 or *I*^2^ > 50% was presented, significant heterogeneity was considered existing among studies, and the random effects model was used to calculate the ORs and 95% CIs; otherwise, the fixed-effects model was applied.^[15,16]^ Funnel plots were also drawn to investigate the potential publication bias of included studies. If funnel plots were distributed approximately symmetrically, results indicated an absence of obvious publication bias. Begg and Egger tests were also carried out to observe the publication bias. Furthermore, subgroup analysis based on sample subtype and race was performed to lower the between-study heterogeneity. Sensitivity analysis was conducted by excluding individual study to assess stability of results, and outlying studies were removed.

## 3 Results

### 3.1 Characteristics of published studies

**Figure 1:**
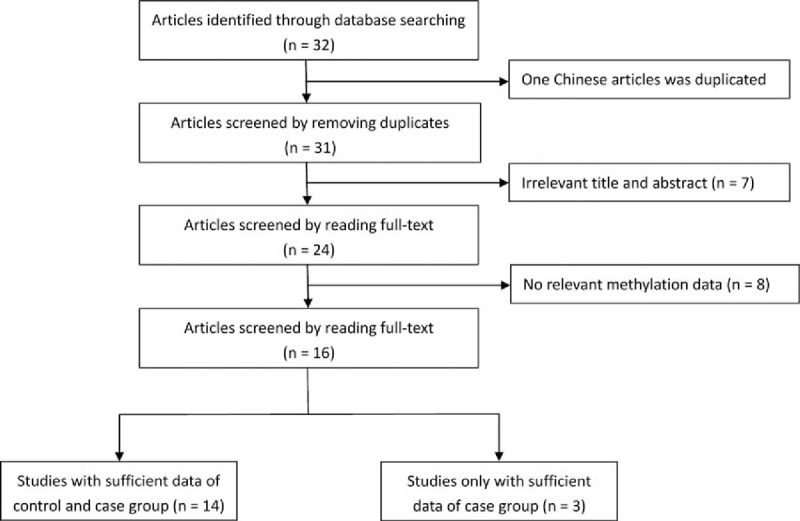
Flow diagram of literature selection.

The original searching yielded a total of 32 publications using the search terms. Thirty-one publications were obtained after 1 duplicated article was removed. Through reading title and abstract, 24 publications were remained. Finally, 16 articles (17 studies) with 1736 cases and 1291 controls were included in this meta-analysis by scanning the full texts. In these publications, 14 studies had sufficient data of both controls and cases group, and 3 studies only had sufficient data of cases. Thus, the remaining 3 studies were used to observe the association between MGMT promoter hypermethylation and clinical characteristics of GC. Moreover, 8 studies considered adjacent tissue as control group, while 4 studies put normal tissue as control group. All studies on hypermethylation of MGMT promoter were genotyped in GC tissue except 2, which used blood sample. On the other hand, 2 studies were in Asians and 15 studies were in Caucasians (Table 1, Fig. 1).

**Table 1:**
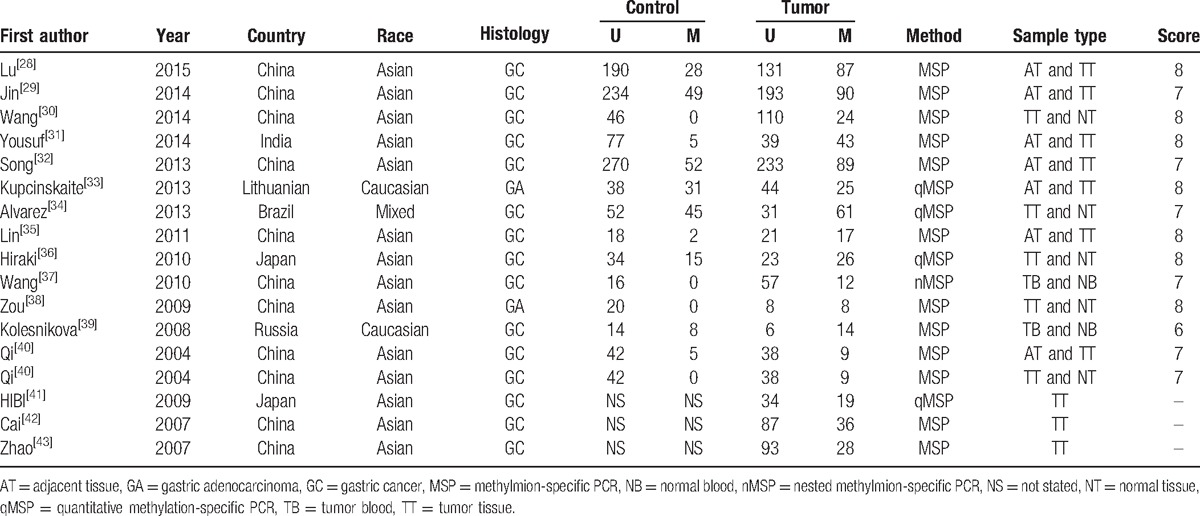
Main characteristics of all eligible studies.

### 3.2 Quantitative analysis

**Figure 2:**
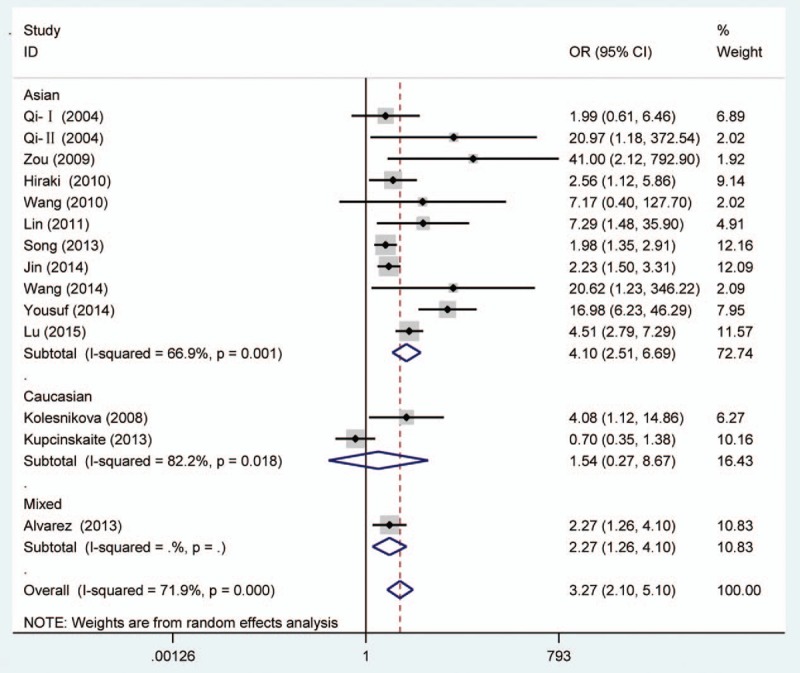
Forest plot of gastric cancer risk associated with O^6^-methyguanine DNA methyltransferase promoter hypermethylation stratified by race. DNA = deoxyribonucleic acid.

**Figure 3:**
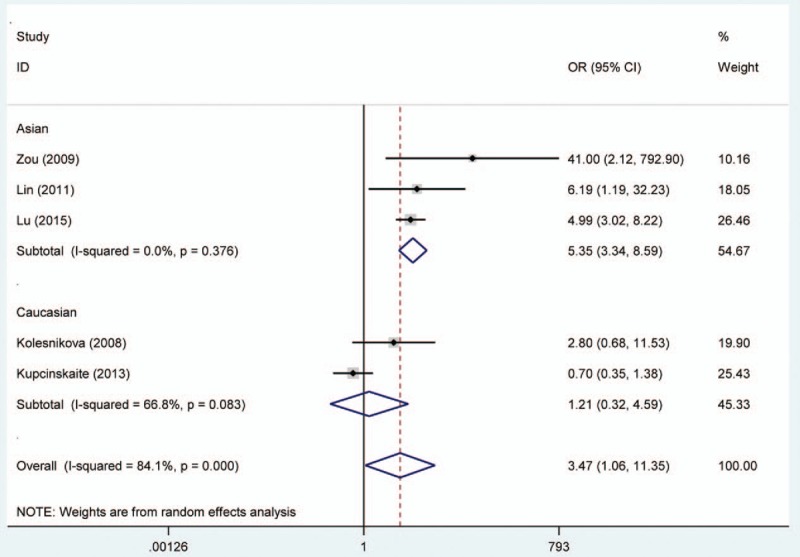
Forest plot of gastric adenocarcinoma risk associated with O^6^-methyguanine DNA methyltransferase promoter hypermethylation stratified by race. DNA = deoxyribonucleic acid.

**Figure 4:**
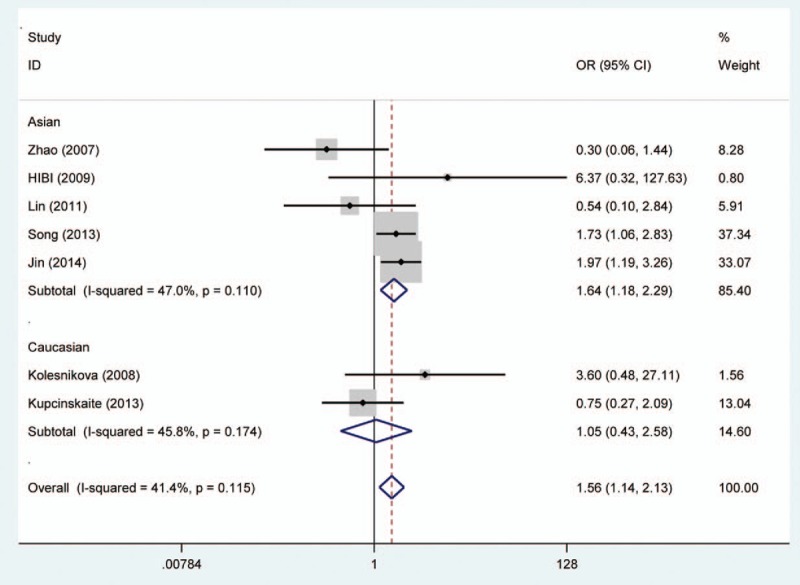
Forest plot of O^6^-methyguanine DNA methyltransferase promoter hypermethylation associated with lymph node metastasis of gastric tumor stratified by race. DNA = deoxyribonucleic acid.

**Figure 5:**
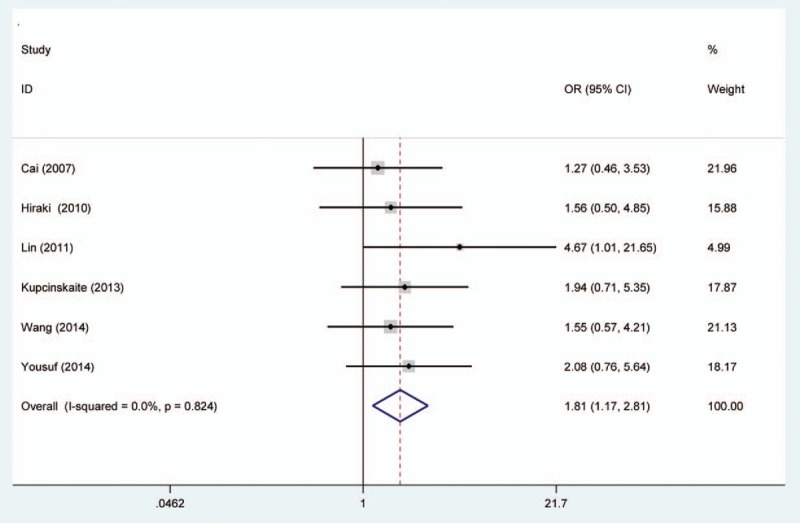
Forest plot of O^6^-methyguanine DNA methyltransferase promoter hypermethylation associated with Lauren classification of gastric cancer. DNA = deoxyribonucleic acid.

**Figure 6:**
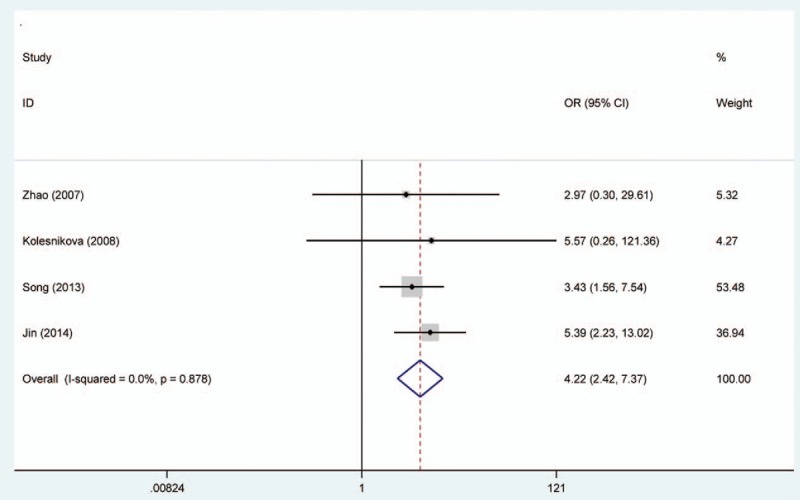
Forest plot of O^6^-methyguanine DNA methyltransferase promoter hypermethylation associated with distant metastasis of gastric cancer. DNA = deoxyribonucleic acid.

**Figure 7:**
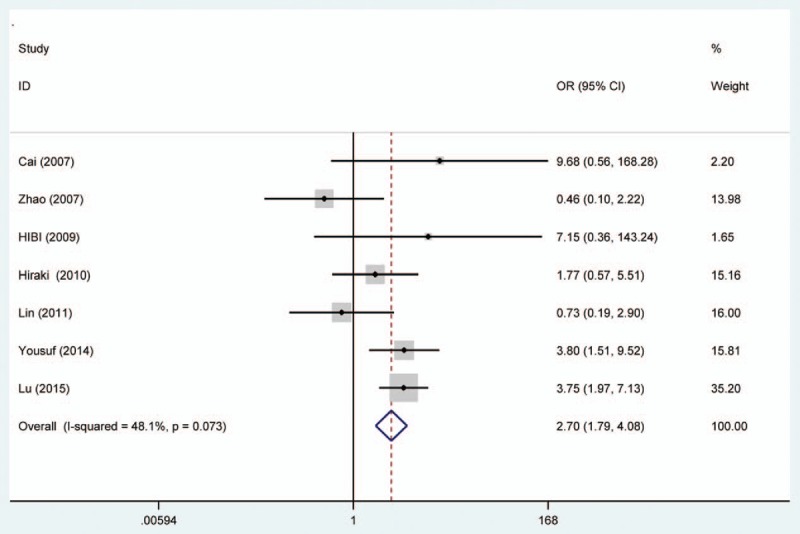
Forest plot of O^6^-methyguanine DNA methyltransferase promoter hypermethylation associated with TNM-stage of gastric cancer. DNA = deoxyribonucleic acid.

Random effects model was applied for the analysis of the association between MGMT promoter hypermethylation and GC risk, since a significant heterogeneity was found (*I*^2^ = 61.9%, *P* = .001). In the subgroup analysis based on sample subtype, the heterogeneity significantly lowered in blood sample and normal tissue (for adjacent tissue, *I*^2^ = 71.5%, *P* = .001; for normal tissue, *I*^2^ = 64.7%, *P* = .037; for blood sample, *I*^2^ = 0.0%, *P* = .715; for total, *I*^2^ = 61.9%, *P* = .001). However, significant associations were still found in the subgroup analysis (for adjacent tissue, OR = 2.44, CI = 1.57–3.50, *P* < .05; for normal tissue, OR = 9.99, CI = 1.60–62.45, *P* < .05; for blood sample, OR = 4.49, CI = 1.38–14.59, *P* < .05). In the subgroup analysis based on GC subtype, the MGMT promoter hypermethylation significantly increased the risk of gastric adenocarcinoma (OR = 3.47, CI = 1.06–11.35, *P* < .05). The results of subgroup analysis based on race, displaying on the forest plot, indicated that the race was one of the sources of heterogeneity. In addition, the frequency of MGMT promoter hypermethylation in M1-stage of GC was significantly higher than those in M0-stage (OR = 4.22, CI = 2.42–7.37, *P* < .05). In a separate analysis, there was a significant association of MGMT promoter hypermethylation with lymph node (LN) metastasis of gastric tumor (OR = 1.56, CI = 1.14–2.13, *P* < .05), especially in Asians (OR = 1.64, CI = 1.18–2.29, *P* < .05). The probability of occurrence of diffuse GC was 1.81 times than that of intestinal GC (OR = 1.81, CI = 1.17–2.81, *P* < .05). In the analysis for TNM-stage, the MGMT promoter hypermethylation accelerated the progress of GC (OR = 2.70, CI = 1.79–4.08, *P* < .05). Moreover, no significant associations of MGMT promoter hypermethylation with tumor differentiation, T-stage, and gender were detected (Figs. 2–7, Table 2).

**Table 2:**
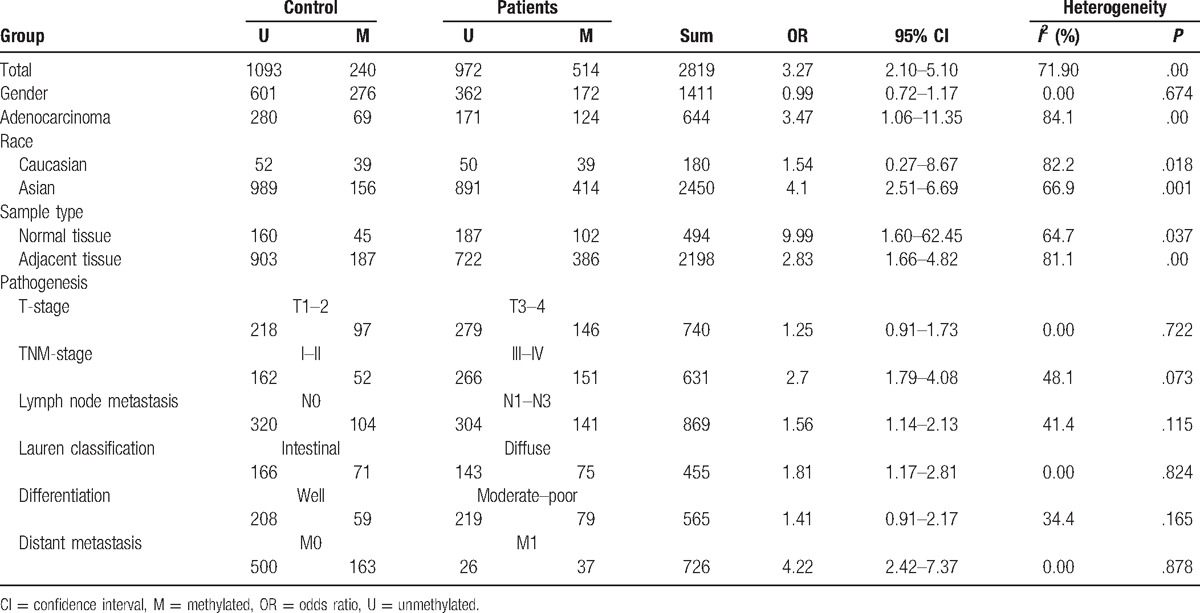
Results of meta-analysis.

### 3.3 Quality assessment

In total, the global quality assessment score, according to the NOS table, ranged from 6 to 8. This result indicated that the literatures of medium and high quality were included in this meta-analysis. This situation increased the accuracy of the results.

### 3.4 Heterogeneity analysis

Although a significant association of MGMT promoter hypermethylation with GC risk was found, heterogeneity among studies was found. Considering the existence of heterogeneity, in order to lower the heterogeneity present in different studies, subgroup analysis based on sample subtype and race was performed in this meta-analysis. Furthermore, meta-regression analysis was also carried out to find the source of heterogeneity (meta-regression analysis for total number of sample, *P* = .55; for adjacent tissue, *P* = .19; for blood, *P* = .55; for normal tissue, *P* = .26; for Asian, *P* = .12; and for Caucasian, *P* = .10). The results indicated that sample type and race were not the main source of heterogeneity, especially in Asians. However, the 2 factors, cancer type and ethnicity, still lowered the heterogeneity among studies according to the subgroup analysis.

### 3.5 Publication bias and sensitivity analysis

**Figure 8:**
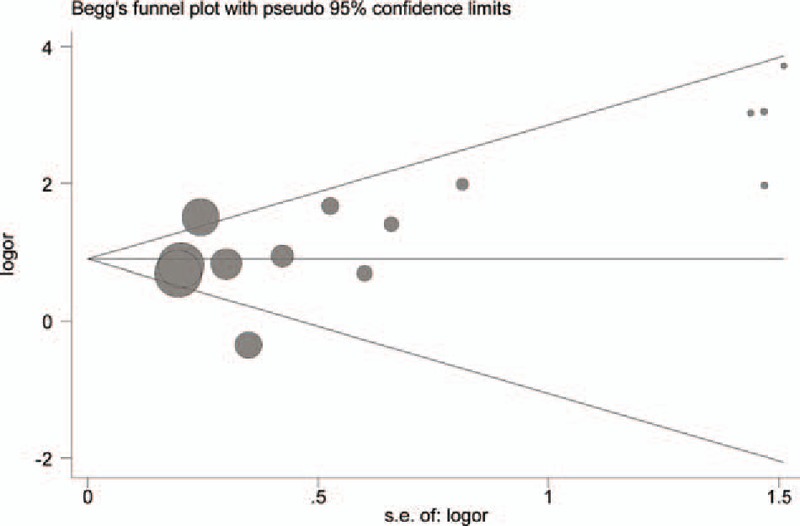
Begg funnel plot for the association between the O^6^-methyguanine DNA methyltransferase promoter hypermethylation and gastric cancer risk. SE = standard error. DNA = deoxyribonucleic acid.

**Figure 9:**
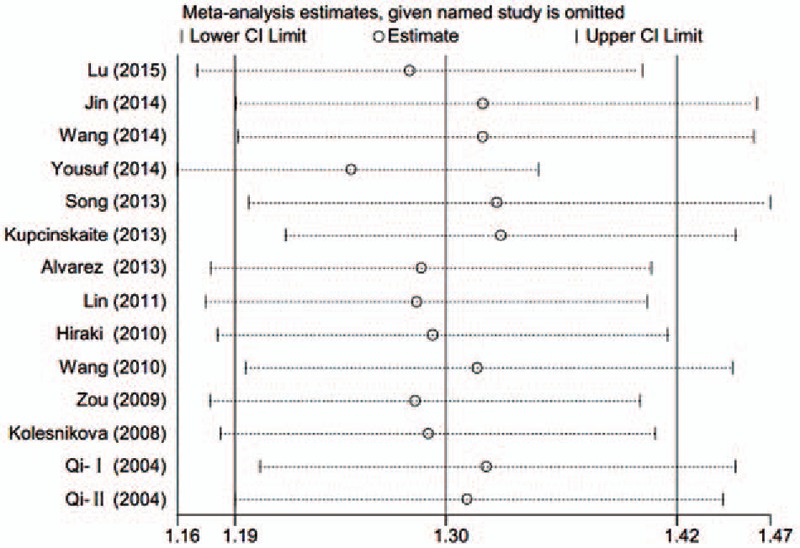
Sensitivity analysis of association between O^6^-methyguanine DNA methyltransferase promoter hypermethylation and gastric cancer risk. DNA = deoxyribonucleic acid.

Begg and Egger tests were conducted to evaluate the publication bias of included literatures. The shapes of the funnel plot and the *P* value did not illustrate any evidence of publication bias (*P* > .05) (Fig. 8). In the meantime, sensitivity analysis indicated that the results were stable (Fig. 9).

## 4 Discussion

Traditional genetic studies on GC have found many gene variations such as single-nucleotide polymorphism, copy number variation, and indel.^[17–19]^ It has been observed that almost half of the tumor suppressor genes were inactivated by gene-promoter hypermethylation in cancers.^[20]^ Therefore, many studies have focused on the discovery of gene-promoter hypermethylation and have found many epigenetic alterations such as *p16INK4a*, *CDKN2B/p151NK2b*, and *p14ARF*.^[21]^ Increasing evidence suggested that different tumors often had different epigenetic alterations such as clustering of gene hypermethylation on the CpG island methylator phenotype. Previous study has found that concordant hypermethylation of multiple locus occurred in 31% GC; however, hypermethylation of independent locus could not satisfactorily predict the risk and prognosis of GC.^[22]^ In fact, no ideal tumor markers for the detection of GC were found in present studies. However, these biomarkers were very important to screen, diagnose, and determine the stage and metastasis of GC. If methylation microarrays could be conveniently used in GC, it might be beneficial for the diagnosis and therapy of GC patients. But before this, a lot of gene methylation alterations should be found and verified in different populations to determine specificity strongly.

Currently, many genes hypermethylated in human cell lines and primary tumors have been found. For instance, promoter hypermethylation of *hMLH1* gene was often found in GCs and had a significantly association with the loss of *hMLH1* expression.^[23]^ Of note, CDH1 promoter hypermethylation had a high frequency in the diffuse histological type. It has been reported that this hypermethylation alteration contributed a lot to LN metastasis of GC.^[24]^*CDH4* gene hypermethylation has also been found in a higher frequency in GC tissue than adjacent mucosa, and it might have a significant association with progression of GC.^[25]^ Furthermore, APC, H-Cadherin, DAPK, RASSF1A, XIAP, VLDLR, RUNX3, RARβ, CASP8, and MGMT had abnormal epigenetic variations in the development of GC.^[26]^ Although previous studies have found that the hypermethylation of *MGMT* gene accounted for 31% in GC patients, the association between MGMT promoter hypermethylation and risk of GC was inconsistent.^[27]^ Thus, this meta-analysis was performed by summarizing the existing data to observe the correlation.

From the forest plots, 3 studies did not show a significant correlation,^[33,37,40]^ while 11 studies observed a significant association of MGMT promoter hypermethylation with risk of GC.^[28–29,31–36,38,39]^ In the calculation of total ORs, the 3 studies contributed to 21.61% of weight; thus, it was necessary to conduct a meta-analysis to determine the association between MGMT promoter hypermethylation and GC risk. From the overall analysis, the MGMT promoter hypermethylation might significantly increase the risk of GC. According to the subgroup of gastric adenocarcinoma, a significant result was also detected, and the carriers of MGMT promoter hypermethylation have 3.47 times higher risk of GC than those of hypomethylation. Furthermore, the frequency of MGMT promoter hypermethylation between males and females did not indicate significant difference (OR = 0.92, CI = 0.72–1.17, *P* > .05). This result showed that the occurrence of MGMT promoter hypermethylation was not related to the gender. Stomach carcinoma might be classified into the diffuse cancer and intestinal cancer. These 2 types of GCs had different pathways and mechanisms. The development of intestinal cancers could be divided into 4 steps: atrophic gastritis, intestinal metaplasia, dysplasia, and GC, while no clear steps of development existed in the diffuse cancer.^[26]^ On the basis of this meta-analysis, different frequencies of MGMT promoter methylation were also found between diffuse and intestinal types. The frequency of MGMT promoter hypermethylation in diffuse type was significantly higher than that in intestinal type. In the included studies on Lauren classification, only 1 study had a same significant result.^[35]^ The significant association of MGMT promoter hypermethylation with distant metastasis and LN metastasis was also observed in the forest plot. However, the 2 significant associations were only detected in Asians, and no between-study heterogeneity was observed. Therefore, the MGMT promoter hypermethylation might encourage the metastasis of gastric tumor in Asians and decrease the survival rate of GC. The result in Caucasians might not be accurate due to the small number of included studies; thus, more large-scale and well designed studies were still needed to be conducted. In the meantime, the GC patients’ number of MGMT promoter hypermethylation in stages III to IV was higher than the number of MGMT promoter hypermethylation in stages I and II. Finally, these results demonstrated that the MGMT promoter hypermethylation had a crucial role in the development of GC. Although heterogeneity was detected in the overall analysis, it was significantly decreased after subgroup analysis and random effects model were conducted. According to the results of meta-regression, ethnicity and cancer subtypes were not the main causes of heterogeneity, and other factors might result in the heterogeneity. Furthermore, no significant publication bias was shown on the basis of Begg and Egger tests. Therefore, although some limitations existed in the meta-analysis, these results might provide a direction that MGMT promoter hypermethylation might contribute a lot to the pathogenesis of GC.

In addition to these positive results, negative results were also shown in this meta-analysis. No significant associations of MGMT promoter hypermethylation with gastric tumor differentiation were found. In previous studies, the association was contradictory in included studies that were conducted in different sample sizes and races. For tumor differentiation, 1 study indicated that MGMT promoter hypermethylation encouraged the tumor differentiation.^[31]^ Therefore, to a large extent, the negative result was same with most studies.

In this meta-analysis, we applied the NOS table to evaluate the methodology quality of literatures. The high scores of studies ensured the efficiency and accuracy of statistical analysis. Finally, subgroup based on sample size, sample type, and race was conducted to reduce the heterogeneity, and the heterogeneity lowered obviously. The heterogeneity decreased a lot after subgroup analysis on the basis of race performed, especially in gastric adenocarcinoma (for total, *I*^2^ = 84.1%, *P* = .000; for Caucasians, *I*^2^ = 66.8%, *P* = .083; for Asians, *I*^2^ = 0.0%, *P* = .376).

Some limitations still existed in this meta-analysis. First, selection bias was inevitable due to restriction to articles in English or Chinese language. Second, although subgroup analysis and meta-regression were conducted, heterogeneity present in different study still existed. This result might be caused by individual difference; GC subtypes; or other environmental factors such as diet, smoking, and work environment. Third, the small sample size of studies involving clinical information restricted the statistical power. Fourth, the studied population in these articles only included Caucasians and Asians, and the Asians accounted for a lot. Hence, these results might not represent the overall tendency in the total crowd. Finally, the accuracy of results might be influenced by the small number of subjects and different sample type. Although the number of subjects was moderate, but it was still small after the subgroup analysis based on race and sample type was performed.

In conclusion, the results of this study indicated that *MGMT* gene-promoter hypermethylation was significantly associated with the risk of GC, especially in Asians and gastric adenocarcinoma. Furthermore, *MGMT* gene-promoter hypermethylation might promote the distant metastasis and LN metastasis of gastric tumor.

## References

[1] Qu Y Dang S Hou P Gene methylation in gastric cancer. Clin Chim Acta 2013;424:53–65. 23669186 10.1016/j.cca.2013.05.002

[2] Ferlay J Soerjomataram I Dikshit R Cancer incidence and mortality worldwide: sources, methods and major patterns in GLOBOCAN 2012. Int J Cancer 2015;136:E359–86. 25220842 10.1002/ijc.29210

[3] Hartgrink HH Jansen EP van Grieken NC Gastric cancer. Lancet 2009;374:477–90. 19625077 10.1016/S0140-6736(09)60617-6 PMC4613761

[4] Piazuelo MB Correa P Gastric cancer: overview. Colomb Med (Cali) 2013;44:192–201. 24892619 PMC4002033

[5] Guilford P Humar B Blair V Hereditary diffuse gastric cancer: translation of CDH1 germline mutations into clinical practice. Gastric Cancer 2010;13:1–0. 20373070 10.1007/s10120-009-0531-x

[6] Abadi AT Taghvaei T Wolfram L Infection with *Helicobacter pylori* strains lacking dupA is associated with an increased risk of gastric ulcer and gastric cancer development. J Med Microbiol 2012;61:23–30. 21903829 10.1099/jmm.0.027052-0

[7] Kang C Song JJ Lee J Epigenetics: an emerging player in gastric cancer. World J Gastroenterol 2014;20:6433–47. 24914365 10.3748/wjg.v20.i21.6433 PMC4047329

[8] Wang Y Leung FC An evaluation of new criteria for CpG islands in the human genome as gene markers. Bioinformatics 2004;20:1170–7. 14764558 10.1093/bioinformatics/bth059

[9] Bird A DNA methylation patterns and epigenetic memory. Genes Dev 2002;16:6–21. 11782440 10.1101/gad.947102

[10] Tan RY Ngeow J Hereditary diffuse gastric cancer. What the clinician should know. World J Gastrointest Oncol 2015;7:153–60. 26380059 10.4251/wjgo.v7.i9.153 PMC4569593

[11] Gerson SL Clinical relevance of MGMT in the treatment of cancer. J Clin Oncol 2002;20:2388–99. 11981013 10.1200/JCO.2002.06.110

[12] Hegi ME Sciuscio D Murat A Epigenetic deregulation of DNA repair and its potential for therapy. Clin Cancer Res 2009;15:5026–31. 19671858 10.1158/1078-0432.CCR-08-1169

[13] Hegi ME Diserens AC Gorlia T MGMT gene silencing and benefit from temozolomide in glioblastoma. N Engl J Med 2005;352:997–1003. 15758010 10.1056/NEJMoa043331

[14] Dunn J Baborie A Alam F Extent of MGMT promoter methylation correlates with outcome in glioblastomas given temozolomide and radiotherapy. Br J Cancer 2009;101:124–31. 19536096 10.1038/sj.bjc.6605127 PMC2713697

[15] DerSimonian R Laird N Meta-analysis in clinical trials. Control Clin Trials 1986;7:177–88. 3802833 10.1016/0197-2456(86)90046-2

[16] Mantel N Haenszel W Statistical aspects of the analysis of data from retrospective studies of disease. J Natl Cancer Inst 1959;22:719–48. 13655060

[17] Thacker J The RAD51 gene family, genetic instability and cancer. Cancer Lett 2005;219:125–35. 15723711 10.1016/j.canlet.2004.08.018

[18] Kim K Park U Wang J Gene profiling of colonic serrated adenomas by using oligonucleotide microarray. Int J Colorectal Dis 2008;23:569–80. 18305945 10.1007/s00384-008-0451-y

[19] Mancino M Strizzi L Wechselberger C Regulation of human Cripto-1 gene expression by TGF-beta1 and BMP-4 in embryonal and colon cancer cells. J Cell Physiol 2008;215:192–203. 17941089 10.1002/jcp.21301

[20] Hinshelwood RA Clark SJ Breast cancer epigenetics: normal human mammary epithelial cells as a model system. J Mol Med 2008;86:1315–28. 18716754 10.1007/s00109-008-0386-3

[21] Milne AN Sitarz R Carvalho R Molecular analysis of primary gastric cancer, corresponding xenografts, and 2 novel gastric carcinoma cell lines reveals novel alterations in gastric carcinogenesis. Hum Pathol 2007;38:903–13. 17376510 10.1016/j.humpath.2006.12.010

[22] Panani AD Cytogenetic and molecular aspects of gastric cancer: clinical implications. Cancer Lett 2008;266:99–115. 18381231 10.1016/j.canlet.2008.02.053

[23] Fleisher AS Esteller M Wang S Hypermethylation of the hMLH1 gene promoter in human gastric cancers with microsatellite instability. Cancer Res 1999;59:1090–5. 10070967

[24] Graziano F Arduini F Ruzzo A Prognostic analysis of E-cadherin gene promoter hypermethylation in patients with surgically resected, node-positive, diffuse gastric cancer. Clin Cancer Res 2004;10:2784–9. 15102685 10.1158/1078-0432.ccr-03-0320

[25] Miotto E Sabbioni S Veronese A Frequent aberrant methylation of the CDH4 gene promoter in human colorectal and gastric cancer. Cancer Res 2004;64:8156–9. 15548679 10.1158/0008-5472.CAN-04-3000

[26] Fu DG Epigenetic alterations in gastric cancer (review). Mol Med Rep 2015;12:3223–30. 25997695 10.3892/mmr.2015.3816 PMC4526033

[27] Jones PA Baylin SB The fundamental role of epigenetic events in cancer. Nat Rev Genet 2002;3:415–28. 12042769 10.1038/nrg816

[28] Lu GW Zhang YX Xia YM The association between MGMT promoter methylation and clinical characteristic of gastric cancer. Mod Pract Med 2015;27:745–54.

[29] Jin J Xie L Xie CH Aberrant DNA methylation of MGMT and hMLH1 genes in prediction of gastric cancer. Genet Mol Res 2014;13:4140–5. 24938706 10.4238/2014.May.30.9

[30] Wang M Li Y Gao J p16 Methylation is associated with chemosensitivity to fluorouracil in patients with advanced gastric cancer. Med Oncol 2014;31:988. 24816738 10.1007/s12032-014-0988-2

[31] Yousuf A Bhat MY Pandith AA MGMT gene silencing by promoter hypermethylation in gastric cancer in a high incidence area. Cell Oncol 2014;37:245–52. 10.1007/s13402-014-0179-3 PMC13004438 25008999

[32] Song B Ai J Kong X Aberrant DNA methylation of P16, MGMT, and hMLH1 genes in combination with MTHFR C677T genetic polymorphism in gastric cancer. Pak J Med Sci 2013;29:1338–43. 24550949 10.12669/pjms.296.3711 PMC3905372

[33] Kupcinskaite-Noreikiene R Skieceviciene J Jonaitis L CpG island methylation of the MLH1, MGMT, DAPK, and CASP8 genes in cancerous and adjacent noncancerous stomach tissues. Medicina (Kaunas) 2013;49:361–6. 24509146

[34] Alvarez MC Santos JC Maniezzo N MGMT and MLH1 methylation in *Helicobacter pylori*-infected children and adults. World J Gastroenterol 2013;19:3043–51. 23716983 10.3748/wjg.v19.i20.3043 PMC3662943

[35] Lin H Cao J Dai WJ Correlation between suppressor genes promoter hypermethylation and chemosensitivity of gastric cancer. Clin Basic Res 2011;18:688–92.

[36] Hiraki M Kitajima Y Sato S Aberrant gene methylation in the lymph nodes provides a possible marker for diagnosing micrometastasis in gastric cancer. Ann Surg Oncol 2010;17:1177–86. 19957042 10.1245/s10434-009-0815-8

[37] Wang Y Zhou L Chen XQ Detection of p16 and MGMT promoter methylation in gastric carcinomas by nested methylation-specific polymerase chain reaction. World Chin J Digestol 2010;18:384–7.

[38] Zou XP Zhang B Zhang XQ Promoter hypermethylation of multiple genes in early gastric adenocarcinoma and precancerous lesions. Hum Pathol 2009;40:1534–42. 19695681 10.1016/j.humpath.2009.01.029

[39] Kolesnikova EV Tamkovich SN Bryzgunova OE Circulating DNA in the blood of gastric cancer patients. Ann N Y Acad Sci 2008;1137:226–31. 18837952 10.1196/annals.1448.009

[40] Qi J Zu YY Yang D The association between CpG methylation of MGMT promoter and loss of protein expression in gastric cancer. World Journal of Gastroenterology 2004;12:751–3.

[41] Hibi K Sakata M Yokomizo K Methylation of the MGMT gene is frequently detected in advanced gastric carcinoma. Anticancer Res 2009;29:5053–5. 20044616

[42] Cai JC Liu D Zhang HP Frequent promoter hypermethylation of several tumor suppressor genes in gastric carcinoma and foveolar epithelium. Chin J Oncol 2007;29:510–3. 18069630

[43] Zhao YF Zhang YG Tian XX Aberrant methylation of multiple genes in gastric carcinomas. Int J Surg Pathol 2007;15:242–51. 17652530 10.1177/1066896907302117

